# Exploring the Efficacy of Benralizumab in Chronic Eosinophilic Pneumonia Following Corticosteroid Therapeutic Failure: A Case Report

**DOI:** 10.7759/cureus.87634

**Published:** 2025-07-09

**Authors:** Sofia De La O Villalobos, Pablo Olivas Melendez, Adriana Saenz Ramirez, Mariana Gonzalez Plascencia, Isai R Castillo Cabrera

**Affiliations:** 1 Internal Medicine, Instituto de Seguridad y Servicios Sociales de los Trabajadores del Estado (ISSSTE) Hospital General Lazaro Cardenas / Autonomous University of Chihuahua, Chihuahua, MEX; 2 Pulmonology, Christus Muguerza Chihuahua / University of Monterrey, Chihuahua, MEX

**Keywords:** anti-il5, benralizumab, chronic eosinophilic pneumonia, covid-19, interstitial pneumonia, nasal polyps, severe eosinophilic asthma, steroid-resistant

## Abstract

Chronic eosinophilic pneumonia is a rare idiopathic disease characterized by eosinophilic infiltrates in the alveoli and interstitial lung spaces. There are no specific diagnostic tests for this condition, but it is suspected in patients presenting with dyspnea, cough, a tomographic pattern of consolidations, peripheral ground-glass opacities, and peripheral eosinophilia. This report presents the case of a male patient with chronic eosinophilic pneumonia that was unresponsive to corticosteroid treatment.

## Introduction

Chronic eosinophilic pneumonia is a rare, idiopathic pulmonary disorder characterized by eosinophilic infiltration of the alveolar spaces and interstitial lung tissue. Although there are no definitive diagnostic tests, the diagnosis is typically considered in patients who present with progressive dyspnea, persistent cough, peripheral blood eosinophilia, and characteristic imaging findings, most notably, peripheral ground-glass opacities and consolidations on chest CT scans. In this report, we present the case of a male patient with chronic eosinophilic pneumonia who exhibited a poor response to standard corticosteroid therapy [1].

## Case presentation

The patient is a 65-year-old male with a history of social alcohol use and a positive smoking history beginning at approximately 18 years of age, averaging three to four cigarettes per day. He ceased smoking 30 years ago. He had been under evaluation for diffuse interstitial lung disease over the past decade and was maintained on home oxygen therapy (2 L/minute for two hours daily), along with pharmacologic management including theophylline, tiotropium bromide, salmeterol/fluticasone, and deflazacort.

A complete blood count (CBC) from 2019 revealed peripheral eosinophilia of 830 cells/µL, with subsequent measurements ranging between 1,300 and 1,500 cells/µL. Based on clinical findings and imaging, a diagnosis of chronic eosinophilic pneumonia with fibrotic changes was established by the pulmonology team. Over the past 10 years, the patient experienced recurrent exacerbations despite ongoing treatment with oral corticosteroids.

In April 2024, while on oral prednisone at a dose of 10 mg daily, the patient continued to report worsening symptoms, including persistent cough and progressive dyspnea. Peripheral eosinophilia remained elevated, prompting further diagnostic workup to exclude other potential causes, such as parasitic infections and systemic vasculitis. Laboratory tests performed on May 20, 2024, revealed the following values: hemoglobin 15.1 g/dL, hematocrit 44.2%, platelet count 350,000/µL, leukocyte count 9.94 × 10⁹/L, neutrophils 5.24 × 10⁹/L, and eosinophils 1.22 × 10⁹/L. A serial stool parasitology study (three samples) was negative. Serologic testing yielded negative results for anti-proteinase 3 antibodies (c-ANCA < 2.00 IU/mL), anti-myeloperoxidase antibodies (p-ANCA < 2.00 IU/mL), antinuclear antibodies (ANA < 1:40), and total IgE measured at 292 IU/mL.

**Figure 1:**
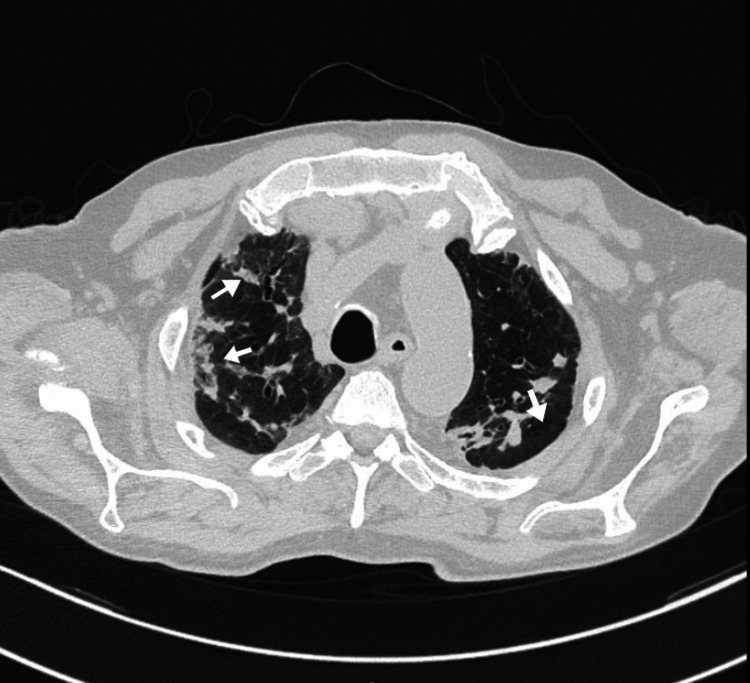
High-resolution computed tomography (HRCT) of the chest findings High-resolution computed tomography (HRCT) of the chest showing ground-glass opacity predominantly distributed in the peribronchovascular and subpleural regions.

**Figure 2:**
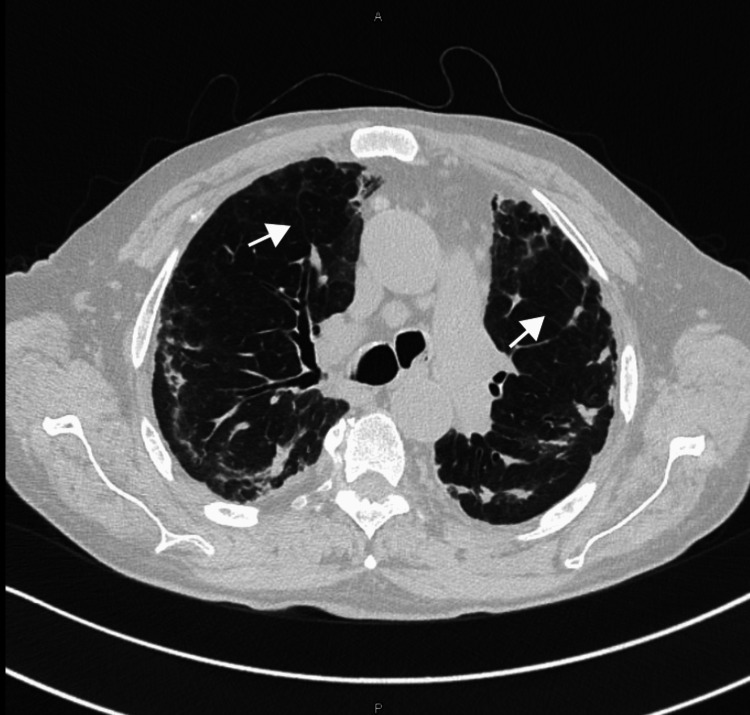
HRCT findings in chronic eosinophilic pneumonia High-resolution computed tomography (HRCT) of the chest showing diffuse mosaic attenuation patterns.

High-resolution computed tomography (HRCT) of the chest, performed with lung parenchyma window settings, revealed peripheral and peribronchovascular ground-glass opacities with associated cicatricial atelectasis. Additional findings included traction bronchiectasis, predominantly in the upper lobes, and diffuse mosaic attenuation throughout the lung fields, findings consistent with chronic fibrotic and inflammatory changes (Figures 1, 2).

Spirometry revealed a forced expiratory volume in one second (FEV₁)/forced vital capacity (FVC) ratio of 109%, with an FEV₁ of 75% and FVC of 69% of predicted values, indicating a restrictive ventilatory pattern. The bronchoalveolar lavage (BAL) analysis demonstrated an eosinophil count exceeding 25%, further supporting the diagnosis of chronic eosinophilic pneumonia.

Given the patient's lack of clinical response to systemic corticosteroids, initiation of anti-interleukin-5 (anti-IL-5) therapy with benralizumab was pursued. This decision was based on multiple criteria, including restrictive spirometry findings, significant eosinophilic infiltration in the BAL fluid, characteristic radiologic features of eosinophilic pneumonia on HRCT, and persistent peripheral eosinophilia despite ongoing corticosteroid treatment.

Following the initiation of benralizumab in June 2024, the patient demonstrated notable clinical improvement. His requirement for home oxygen therapy decreased significantly, and he maintained an oxygen saturation of 94% on room air during follow-up. Repeat laboratory testing confirmed resolution of peripheral eosinophilia, indicating a positive therapeutic response (Table 1).

**Table 1:** Hematologic profile before and after benralizumab treatment A comparison of hematologic parameters obtained before and after the initiation of benralizumab therapy illustrates the patient's clinical response. The initial laboratory results, dated June 26, 2024, represent the baseline profile, which was notable for marked peripheral eosinophilia and mild anemia. In contrast, follow-up laboratory findings from August 6, 2024, demonstrated significant hematologic improvement, including normalization of hemoglobin levels and complete resolution of eosinophilia. These changes are consistent with an effective therapeutic response to anti-IL-5 treatment.

Parameter	June 26, 2024 (Pre-treatment)	August 6, 2024 (Post-treatment)	Reference Range
Hemoglobin (g/dL)	7.2	14.9	14.0–18.0
White blood cells (×10⁹/L)	6.29	5.34	4.4–10.5
Neutrophils (×10⁹/L)	3.38	2.82	2.1–6.1
Lymphocytes (×10⁹/L)	1.68	1.98	1.3–2.9
Monocytes (×10⁹/L)	1.68	0.53	0.3–0.8
Eosinophils (×10⁹/L)	0.28	0.00	0.0–0.2
Basophils (×10⁹/L)	4.50	0.20	0.0–0.1

## Discussion

Chronic eosinophilic pneumonia is a rare idiopathic pulmonary disorder characterized by the accumulation of eosinophils within the interstitial and alveolar spaces of the lungs. The currently accepted diagnostic criteria include (1) persistent respiratory symptoms lasting longer than two weeks, such as cough, dyspnea, or systemic manifestations; (2) abnormal chest imaging findings, typically demonstrating peripheral or upper lobe-predominant infiltrates; (3) evidence of eosinophilic inflammation, supported by one or more of the following: BAL fluid with eosinophils exceeding 25%, peripheral blood eosinophilia, or histopathological confirmation of eosinophilic infiltration in lung tissue; and (4) exclusion of alternative causes of eosinophilic lung disease, including infections, drug reactions, and systemic vasculitides [2].

Systemic corticosteroids remain the mainstay of treatment, often resulting in rapid clinical and radiologic improvement. However, frequent relapses are common and may necessitate prolonged therapy. Long-term corticosteroid use is associated with significant adverse effects, including an increased risk of opportunistic infections, osteoporosis-related fractures, and gastrointestinal complications such as peptic ulcer disease [3].

Benralizumab, a monoclonal antibody targeting the interleukin-5 receptor alpha (IL-5Rα), has demonstrated efficacy in depleting eosinophils through antibody-dependent cell-mediated cytotoxicity (ADCC). This mechanism provides a corticosteroid-sparing alternative for patients with eosinophilic disorders who are refractory to standard treatment [4,5].

## Conclusions

In this case, following the initiation of benralizumab, there was complete resolution of eosinophilia and normalization of hemoglobin levels, indicating a robust hematologic response to therapy. Clinically, the patient experienced resolution of symptoms and a significant reduction in supplemental oxygen requirements, further supporting effective disease control. The normalization of monocyte and basophil counts also suggests a broader reduction in systemic inflammation. Collectively, the hematologic profile reinforces the conclusion of effective disease control following treatment.

Given the substantial side effects associated with long-term corticosteroid use, benralizumab represents a valuable alternative for patients who have failed corticosteroid therapy or are at risk for complications related to prolonged corticosteroid exposure. It offers the potential to improve clinical outcomes by effectively reducing eosinophilic activity while minimizing systemic corticosteroid burden.
